# Silencing efficacy prediction: a retrospective study on target mRNA features

**DOI:** 10.1042/BSR20140147

**Published:** 2015-03-31

**Authors:** Devis Pascut, Giorgio Bedogni, Claudio Tiribelli

**Affiliations:** *Italian Liver Foundation, Centre for Liver Studies, Area Science Park Basovizza Bldg Q Ss14 Km 163.5 34149, Trieste, Italy; †Department of Medical, Surgical and Health Sciences, University of Trieste, Strada di Fiume 447, 34149, Trieste, Italy

**Keywords:** prediction, ribonucleic acid (RNA) secondary structure, silencing, siRNA, b-loop, bulge loop, CI, confidence intervals, H, high siRNA efficacy, H-loop, hairpin loop, int-loop, internal loop, L, low siRNA efficacy, M, medium siRNA efficacy, mb-loop, multi-branch loop, mfe, minimum free energies, one-bb, one base bulge, OR, odd ratios, RBP, RNA-binding protein, RISC, RNA-induced silencing complex, VH, very high siRNA efficacy

## Abstract

Post-transcriptional gene silencing is a widely used method to suppress gene expression. Unfortunately only a portion of siRNAs do successfully reduce gene expression. Target mRNA secondary structures and siRNA-mRNA thermodynamic features are believed to contribute to the silencing activity. However, there is still an open discussion as to what determines siRNA efficacy. In this retrospective study, we analysed the target accessibility comparing very high (VH) compared with low (L) efficacy siRNA sequences obtained from the siRecords Database. We determined the contribution of mRNA target local secondary structures on silencing efficacy. Both the univariable and the multivariable logistic regression evidenced no relationship between siRNA efficacy and mRNA target secondary structures. Moreover, none of the thermodynamic and sequence-base parameters taken into consideration (H-b index, ΔG°_overall_, ΔG°_duplex_, ΔG°_break-target_ and GC%) was associated with siRNA efficacy. We found that features believed to be predictive of silencing efficacy are not confirmed to be so when externally evaluated in a large heterogeneous sample. Although it was proposed that silencing efficacy could be influenced by local target accessibility we show that this could be not generalizable because of the diversity of experimental setting that may not be representative of biological systems especially in view of the many local protein factors, usually not taken into consideration, which could hamper the silencing process.

## Abstract 2

We analysed several siRNA-mRNA target features involved in silencing efficacy. We found out that features believed to be predictive of silencing efficacy are not such when transferred to a larger dataset of experiments and different experimental settings.

## INTRODUCTION

RNAi is an evolutionally conserved sequence-specific, post-transcriptional gene silencing mechanism first described in *Caenorhabditis elegans* by Mello and Fire [1]. Long dsRNAs are processed by Dicer, an RNase type III enzyme and cleaved into small fragments of 21–23 bps [2–5]. Such cleavage products are loaded on to the RNA-induced silencing complex (RISC) [6], the passenger strand of dsRNA is cleaved and discarded whereas the guide strand is targeted to the complementary mRNA [6] resulting in its degradation [1,7,8]. RNA interfering is a widely used tool to suppress gene expression but only a portion of siRNAs are able to reduce gene expression [9,10]. There is still an open discussion regarding what determines the silencing efficacy of a specific siRNA. A role may be played by siRNA sequence features [9], siRNA chemical modifications [11], i.e. phosphorylation, interaction with RNA-binding proteins (RBPs) [12,13] and mRNA target accessibility. Several reports point out the relevance of targeted secondary structures for silencing efficacy [14,15]. However, most of the features believed to be relevant for siRNA efficacy were inferred from restricted experimental settings, which may misrepresent their contribution [16,17]. The siRecords database [18] contains more than 17000 records of experimentally validated mammalian siRNAs obtained from about 6000 independent studies. In the present study, siRNA efficacy is categorized as very high (VH; 90%–100% of silencing efficacy), high (H; 70%–90%), medium (M; 50%–70%) and low (L; 0%–50%). In the present retrospective study, we compared 150 VH and 150 L randomly chosen siRNA sequences obtained from different experimental settings to evaluate the role of mRNA targeted secondary structures and siRNA-mRNA thermodynamic features on siRNA efficacy.

## MATERIAL AND METHODS

### siRNA selection and mRNA secondary structure targeting

Three-hundred siRNA sequences targeting 267 human genes were randomly selected from the siRecords database [18]. The 19–21 bp synthetic oligonucleotides were classified as VH (*n*=150) and L (*n*=150) on the basis of silencing efficacy. The gateway for the siRecords database is http://sirecords.biolead.org/index.php. From each siRNA, the corresponding mRNA target sequence was obtained from the NCBI nt database and folded by using the Mfold web server version 2.3 [19] with default settings (http://mfold.rna.albany.edu/?q=mfold). Minimum free energies (mfe) were predicted. Each siRNA sequence was manually aligned to the correspondent global mRNA secondary structure to discriminate the local targeted site among loop, 5′-loop, 3′-loop, internal loop (int-loop), multi-branch loop (mb-loop), H-loop, bulge loop (b-loop), one base bulge (one-bb) and stem structures.

For each siRNA duplex, the number of bases targeting each mRNA local structure and the number of consecutive unpaired bases in the target site were identified.

### H-b index

To determine the overall probability of nts within the siRNA targeting region to form double-stranded complex with other parts of the mRNA, we calculated the H-b index for each pair of siRNA-mRNA. The H-b index is the average number of hydrogen bonds formed in all possible mRNA secondary structures as predicted by the Mfold software. It was proposed by Luo and Chang [20] as a parameter that takes into account the overall contribution of mRNA secondary structures in the siRNA binding. Low values of H-b index indicate that most nts within the target region are in single-stranded structures and are more likely to be accessible by the RISC–siRNA complex.

Each mRNA sequence was previously folded by MFold web server version 2.3 [19] using default settings. The ss-count output was taken into account in order to calculate the H-b index for the siRNA targeted region. The ss-count is the propensity of a base to be single-stranded, as measured by the number of times it happens to be single-stranded in a group of predicted RNA structures. The H-b index was calculated for the two groups of siRNAs according to Luo and Chang [20].

### Binding affinity

The Oligowalk program [21] included in the software package RNAstructure version 5.3 [22] was used to predict the binding affinity of each oligonucleotide to its mRNA target. In particular, three parameters were taken into account:

ΔG°_overall_: The net ΔG in kcal/mol (1 cal≡4.184 J) of oligo-target binding, when all contributions are considered, including breaking target structure and oligo-self-structure, if any. A more negative value indicates tighter binding.

ΔG°_duplex_: The free energy change due to hybridization at the binding site. It measures the oligo-target binding affinity from unstructured states. A more negative value indicates more stable duplex.

ΔG°_break-target_: The free energy cost for opening bps in the region of complementarity to the target so that the binding site becomes completely open. A more negative value indicates less accessible siRNA.

### GC content analysis

The siRNA were separated into siRNA with GC content <25%, between 25% and 55% and >55%. We also evaluated subgroups with GC content from 25% to 34%, 35% to 44%, 45% to 54% and 55 to 64%.

### Statistical analysis

The separate contribution of loop 5′-loop 3′-int-loop, mb-loop, h-loop, b-loop, one-bb and stem to siRNA efficacy was evaluated by univariable logistic regression. Multivariable logistic regression was used to quantify the joint contribution of the same predictors to siRNA efficacy. siRNA efficacy was coded as 0=low and 1=very high and all predictors were modelled as continuous (number of occurrences).

Univariable logistic regression was used to evaluate the contribution of consecutive unpaired bases on the RNA target to the siRNA efficacy. Odd ratios (OR) and robust 95% confidence intervals (CI) were calculated as measures of effect size.

Between-group comparisons were performed with Student's unpaired *t*test for Gaussian distributions and with Mann–Whitney U-test for non-Gaussian distribution.

Fisher's exact test was used to estimate the probability of getting the observed data under the assumption that the frequencies of VH and L siRNA with GC content 25%–55% are the same.

Pearson's chi-square test was used to estimate the probability of getting the observed data under the null hypothesis that the proportions of VH and L siRNA within the GC content subgroups are the same.

## RESULTS AND DISCUSSION

### siRNA efficacy and local secondary structures

The efficacy of siRNA on gene silencing varies within targeting sites of the same mRNA [5,10,23–25]. There is still an open discussion as to what determines those silencing efficacy. Although it was proposed that silencing efficacy could be influenced only by siRNA intrinsic characteristics [9]; previous studies have shown that local target accessibility might contribute to silencing efficacy [14,26]. However, these studies may not be generalizable because they involved low numbers of siRNAs and target structures. The contribution of target accessibility in a given experimental setting may not to be representative of biological systems, especially in view of the many local factors, such as RBPs, chaperons, RNA–RNA interactions, metabolites and ions that have effect on the RNA folding *in vivo* [12,13,27]. In this retrospective study, we analysed the target accessibility and the thermodynamic properties of a large number of siRNA with particular attention to the portion of siRNA targeting a given local secondary structure on the mRNA.

We evaluated 300 randomly chosen siRNA sequences and their targets from siRecords Database. To reduce bias, no more than two siRNAs per working group were selected in each category.

**Figure 1:**
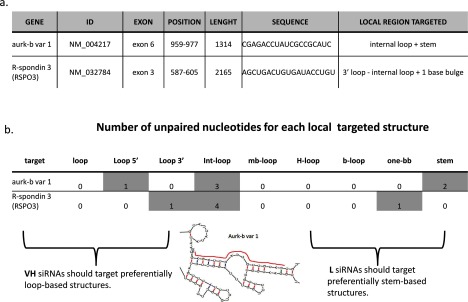
mRNA target secondary structures analysis (**a**) For each siRNA, information on the targeted region within the mRNA was collected. (**b**) The number and position of unpaired bases within the mRNA local targeted region were listed. A single siRNA can target several local structures.

Each mRNA was folded using the Mfold web server version 2.3 [19] and in each siRNA targeted region were identified both the local structures and the number of unpaired bases within the mRNA (see Figure 1 for an example).

Schubert et al. [14] observed that silencing is greatly influenced by the number of paired nts within the mRNA target. These nts are likely to be incorporated in hairpin structures which are unfavourable for siRNA silencing [14,28].

**Figure 2:**
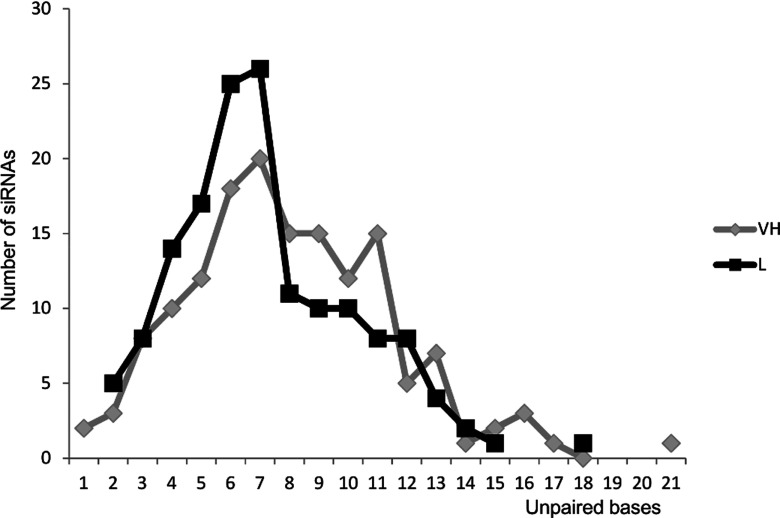
Number of unpaired bases per local mRNA targeted structure

**Figure 3:**
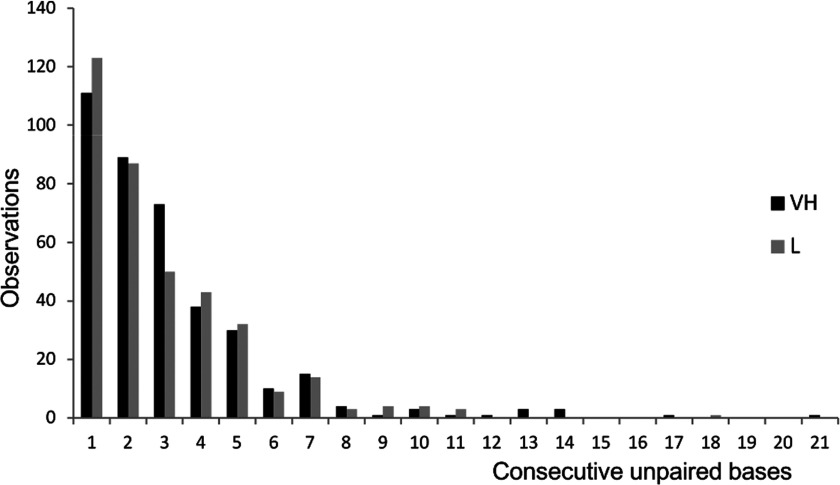
Number of consecutive unpaired bases within the targeted mRNA local structure

To determine whether silencing efficacy depends on the number of unpaired bases within the mRNA targeted region, we evaluated the frequency of siRNAs targeting mRNA sites with the predicted number of base-pairing (Figure 2). Logistic regression was used to evaluate the association between the number of unpaired bases within the mRNA target region and siRNA efficacy. Although most L siRNAs are reported to preferentially target mRNA regions with a low number of unpaired bases (4–8) our analysis showed no association between the number of unpaired bases in the mRNA target region and the siRNA efficacy (OR=1.00; 95% CI, 0.89–1.12; *P*=1.0). We also found no association between the number of consecutive unpaired nts per targeted local mRNA secondary structure (OR=1.03, 95% CI, 0.97–1.09 *P*=0.269; Figure 3). Thus, we were not able to replicate Schubert's findings on an external and much larger dataset.

The position of unpaired nts within the target was taken into account to test whether the mRNA has a role in determining siRNA efficacy. Table 1 summarizes the distribution of unpaired bases within the siRNA targeted structures. It should be noted that a single siRNA can target one, two or more secondary mRNA structures.

**Table 1:** Distribution of unpaired bases within the siRNA targeted structures The table reports the number of observations (siRNAs) targeting mRNA regions with a variable number of unpaired bases. A single siRNA can target more than one local secondary structure within the mRNA. Only few siRNAs (both VH and L) bind to mRNA regions with higher amounts of unpaired nts.

	Loop 5′		Loop 3′		Int-loop		mb-Loop		H-loop		b-Loop		One-bb		Stem	
Unpaired bases	VH	L	VH	L	VH	L	VH	L	VH	L	VH	L	VH	L	VH	L
Number of observations (siRNAs)																
0	94	91	102	111	82	88	83	95	114	118	127	135	99	106	90	89
1	7	18	2	10	1	–	22	18	–	–	1	–	38	41	28	33
2	8	13	10	10	21	17	15	17	–	–	7	3	5	2	15	19
3	8	10	13	5	13	11	9	5	3	6	5	3	–	–	6	2
4	5	5	6	7	7	9	6	4	4	6	2	4	–	–	4	5
5	8	4	4	2	6	10	2	4	13	12	1	2	–	–	–	–
6	5	–	1	–	4	5	1	1	22	2	–	1	–	–	–	1
7	–	–	–	–	–	–	–	–	3	3	–	–	–	–	–	–
8	–	–	–	–	–	–	–	–	2	–	–	–	–	–	–	–
9	–	–	–	–	–	–	–	–	–	–	–	–	–	–	–	–
10	–	1	1	1	–	2	–	–	–	–	–	–	–	–	–	–
11	–	2	–	–	–	1	–	–	–	–	–	–	–	–	–	–
12	–	–	–	–	–	–	1	–	–	–	–	–	–	–	–	–
13	1	–	–	–	1	–	–	–	–	–	–	–	–	–	–	–
14	1	–	1	–	–	–	–	–	1	–	–	–	–	–	–	–
15	–	–	–	–	–	–	–	–	–	–	–	–	–	–	–	–
16	–	–	–	–	–	–	–	–	–	–	–	–	–	–	–	–
17	–	–	1	–	–	–	–	–	–	–	–	–	–	–	–	–
18	–	–	–	1	–	–	–	–	–	–	–	–	–	–	–	–

Since it has been reported that targeting loop-based structures has a positive influence on silencing efficacy [15,20], an association between silencing efficacy and local targeted mRNA structures was expected. However, we found no association between silencing efficacy and targeted mRNA local structure (Table 2). Loop-based structures have been reported to be more favourable for highly effective siRNA binding [15], but we found again that they were not predictive of silencing efficacy (Table 2).

**Table 2:** Statistical analysis

Univariable logistic regression								
	Loop 5′	Loop 3′	Int-loop	mb-Loop	H-loop	b-Loop	One-bb	Stem
OR (95%CI)	1.034 (0.945–1.143)	1.06 (0.95–1.184)	0.983 (0.896–1.079)	1.049 (0.931–1.182)	1.022 (0.928–1.124)	0.944 (0.771–1.155)	1.208 (0.794–1.838)	0.956 (0.768–1.191)
Multivariable logistic regression (all predictors modelled simultaneously)								
	Loop 5′	Loop 3′	Int-loop	mb-Loop	H-loop	b-Loop	One-bb	Stem
OR (95%CI)	1.083 (0.973–1.206)	1.11 (0.979–1.259)	1.067 0.943–1.207)	1.112 (0.96–1.29)	1.089 (0.971–1.222)	1.024 (0.818–1.282)	1.547 (0.963–2.484)	1.08 (0.835–1.398)

Although mRNA secondary structures could influence siRNA binding, they cannot be considered the only factor determining silencing efficacy. Indeed, Holen et al. [10] reported variable silencing efficacies in siRNAs targeting similar predicted secondary structures. Gredell et al. [15] noticed that siRNA silencing efficacy may depend on the transfected cell line independently of the mRNA local region targeted. Furthermore, whereas Overhoff et al. [26] found an improvement in siRNA efficacy based on target accessibility, they also noted that not all siRNAs against inaccessible targets are ineffective. Finally Amarzguioui et al. [29] encountered some discrepancies between silencing experiments conducted *in vitro* and *in vivo*. Putting these observations together with our findings, it appears that the target secondary structure has not a clear role in siRNA silencing efficacy. Other factors, rarely taken into consideration, for instance RBPs, may also influence siRNA binding and silencing efficacy [13]. *In vivo* RNA is not a ‘naked’ molecule, but is bound to a dynamic set of RBPs that begin to be deposited on to the RNA molecule co-transcriptionally [12]. Thus RBPs not only affects the RNA secondary structure but also can occasionally mask siRNA target sites. The implication is that RBPs could influence siRNA efficacy [13]. The full complement of proteins associated *in vivo* is likely to be different from that bound to the same RNA *in vitro.* Frequently *in vitro* experiments are not able to reproduce such a complex system producing biased results and a lack of correlation between *in vitro* accessibility and *in vivo* efficacy [13,29].

### H-b index

One important issue to be considered is the RNA secondary structure prediction. Although there are some evidences about the accuracy of the computational methods [15,26], it is worth to notice that the secondary-structure predictions based on the mfe calculation, used by Mfold, assume that the total free energy is the sum of independent contributions by the singles paired or unpaired nts within the RNA sequence. These contributions could be sensitive to small changes in the energy parameters, temperature, ions concentrations. Not necessarily the thermodynamically most stable structure is the one encountered *in vivo* [12]. Taking into consideration these evidences, we used the H-b index to predict target accessibility [20]. This index takes into account all possible secondary structures of a given RNA to estimate the probability that a base within the RNA sequence is in a single- or double-stranded conformation. If the target region of the mRNA has a more loosened structure (i.e., less intramolecular hydrogen bonding) as in the case of loop-based structures, it should be easier for the siRNA to bind with the targeted mRNA through base-pairing. Thus, the H-b index provides a measure of mRNA accessibility.

Luo and Chang [20] found that low H-b indexes (<25) were predictive of high silencing efficacy. However, they considered a low number of siRNAs and mRNA targets and their data were obtained from a single-group study targeting a single mRNA. In the present study, we found that the mean (S.D.) H-b index was nearly the same for VH compared with L siRNA (31.29±6.97 and 31.82±5.29 respectively, *P*=0.82). Using a cut-point of 25 for the siRNA index, was again found no association with silencing efficacy (95%CI, 0.88–1.9, *P*=0.085).

### Binding affinity

Several groups recommend considering thermodynamic properties when performing siRNA studies [21,30]. In the present work, we used Oligowalk v3.5 [21] to analyse the thermodynamic properties of siRNAs. In detail, we evaluated overall ΔG°, i.e. the overall Gibbs free energy change of RNA binding at 37°C. This parameter takes into account the ΔG° variation of breaking target structures and of intramolecular secondary structural formation, including self-structure formation in the target and in the siRNA. Since the silencing efficacy should be positively associated with the stability of the siRNA-mRNA duplex and negatively associated with the stability of the siRNA and mRNA secondary structure, the overall ΔG° should be associated with siRNA efficacy. However, we found that overall ΔG° was similar in VH compared with L siRNA [mean (S.D.) −19.18±7.37 and −18.26±7.09 respectively; *P*=0.27]. It appears therefore that ΔG°_overall_ is not associated with siRNA efficacy although some reports have shown a weak association [30]. Similar results were obtained when considering the ΔG°_break-target_ that corresponds to the free energy cost for breaking the target intramolecular bps at the binding site that becomes completely single stranded. More negative values mean that the binding site is less accessible for siRNA binding.

In a sample of about 100 siRNAs challenged against three human genes, Shao et al. [31] found that ΔG°_break-target_ was the only predictor of siRNA efficacy. In the present study, based on 300 siRNAs targeting 267 different mRNAs, we find similar ΔG°_break-target_ values for VH compared with L siRNAs [mean (S.D.) −14.56±8.52 compared with −14.58±7.04; *P*=0.99].

We also evaluated the stability of the potential siRNA-mRNA duplex (ΔG°M_duplex_). In theory, the more stable is the duplex, the more negative is the ΔG°_duplex_ value. Although we found a statistically significant difference in ΔG°_duplex_ between VH and L siRNA, (−35.57±4.73 and for L −33.11±10.82; *P*=0.04), this difference is unlikely to be biologically relevant. A poor predictive ability for ΔG°_duplex_ was reported also from Shao et al. [31]. An association between ΔG°_duplex_ and siRNA efficacy was reported by Matveeva et al. [30] at a cut-off −30 kcal/mol. However, use of such cut-off offered no improvement in the present study as the percentage of L siRNA with ΔG°_duplex_ ≤ −30 kcal/mol was actually greater than VH siRNAs (14.7% compared with 8.7%; *P*=0.09).

### GC content and siRNA efficacy

Some studies have shown that a low GC content (30%–55%) has a positive effect on silencing [32–34] whereas other studies have shown only a weak association [35,36]. CG content surely influences both RISC loading and target affinity and specificity. A higher CG content may negatively influence the dissociation of the siRNA duplex hampering RISC loading [37,38]. On the other side, it has been postulated that a low GC percentage may decrease silencing by reducing target affinity [39]. In a previous study [40] where several CG content ranges were analysed, 51.7% of effective siRNA (product level less than 30%) had a GC content of 25%–55%. When shifting this range toward higher GC contents (35%–75%) the percentage of effective siRNAs reduced up to 42.2%. In our analysis we considered also the threshold 25%–55% and we did not find any statistically significant difference between VH and L siRNA (86% compared with 79%; *P*=0.2) indicating that this CG range is not a discriminating factor for VH and L siRNAs. To support these results, we tested the null hypothesis that the 51.7% of VH siRNA have a GC content 25%–55% obtaining a statistically significant difference between observed and expected frequencies (*P*=0.001). When considering different subgroups (25%–34%; 35%–44%; 45%–54%; 55%–64%) again we did not find any statistically relevant difference among VH and L siRNAs (8% compared with 12%; 36% compared with 28%; 46.7% compared with 43%, 14% compared with 14%; *P*=0.85). Although GC% affects the thermodynamic stability of siRNA duplex and target binding, at least *in vitro* and *in silico*, it is not central for silencing efficacy *in vivo* and this is in agreement with the findings of Tafer et al. [41] who consider the GC content a poor predictor of siRNA efficacy.

## CONCLUSION

The evidence that siRNAs synthesized against a common RNA target have different silencing efficacies has pointed out the importance of secondary structures on silencing activities. Studies have shown that a relationship between siRNA efficacy and mRNA target secondary structure exists [15,26]; however, a common drawback of these studies is the so-called ‘over-fitting problem’ [16,17]. In other words, the siRNA-mRNA target features involved in siRNA efficacy extracted from data that have small sample size and unique experimental settings (i.e. a set of siRNA against the same target or a restrict number of targets) are likely to perform unsatisfactorily when applied on large datasets under different experimental settings. *In vitro* experiments could not accurately represent the dynamic setting encountered *in vivo*. This is due to the presence of RBPs, chaperons, RNA–RNA interactions, metabolites and ions that affect the RNA folding [12]. In addition, differences in the expression and turnover of several components of the RISC pathways were observed among cell lines and tissues [27,42]. Another aspect influencing the degree to which a target gene is silenced *in vitro* is represented by the target protein stability and turnover. It is accepted that many proteins have different cellular stabilities depending on their biological functions. Poor correlation between mRNA and protein, as expected for transcription factors, cell-cycle modulators and signalling transducers, could provide a biased evaluation of siRNA efficacy [43].

In the present study, we analysed several siRNA-mRNA target features involved in silencing efficacy, most of them derived from a unique experimental setting. We found out that features believed to be predictive of silencing efficacy are not such when transferred to a larger dataset of experiments and different experimental settings. In particular, from our analysis we cannot derive any preference for VH and L siRNAs in targeting a particular mRNA local structure. Moreover none of the considered thermodynamic and sequence-base parameters (i.e. ΔG°_overall_, ΔG°_duplex_, ΔG°_break-target_ and GC%) is predictive for siRNA efficacy. We believe that although secondary structures and thermodynamic parameters are important for siRNA efficiency, they are not sufficient to reliably predict siRNA efficacy [44], many other factors could hamper the silencing process *in vivo* preventing mRNA target recognition and binding [12,13]. Usually not taken into consideration this aspect could be relevant since, for instance, mRNA interacting proteins are not the same for all RNAs and they change depending on the RNA processing stage [45]. Thus the question as to which factors determine siRNA silencing efficacy is still open and should be reconsidered in view of these observations.
